# Improvements of insulin resistance in ovariectomized rats by a novel phytoestrogen from *Curcuma comosa *Roxb

**DOI:** 10.1186/1472-6882-12-28

**Published:** 2012-03-30

**Authors:** Mujalin Prasannarong, Vitoon Saengsirisuwan, Pawinee Piyachaturawat, Apichart Suksamrarn

**Affiliations:** 1 Department of Physiology, Faculty of Science, Mahidol University, Rama VI Road, Bangkok 10400, Thailand; 2 Department of Chemistry, Faculty of Science, Ramkhamhaeng University, Bangkok 10240, Thailand

## Abstract

**Background:**

*Curcuma comosa *Roxb. (*C. comosa*) is an indigenous medicinal herb that has been used in Thailand as a dietary supplement to relieve postmenopausal symptoms. Recently, a novel phytoestrogen, (3*R*)-1,7-diphenyl-(4*E*,6*E*)-4,6-heptadien-3-ol or compound 049, has been isolated and no study thus far has investigated the role of *C. comosa *in preventing metabolic alterations occurring in estrogen-deprived state. The present study investigated the long-term effects (12 weeks) of *C. comosa *hexane extract and compound 049 on insulin resistance in prolonged estrogen-deprived rats.

**Methods:**

Female Sprague-Dawley rats were ovariectomized (OVX) and treated with *C. comosa *hexane extract (125 mg, 250 mg, or 500 mg/kg body weight (BW)) and compound 049 (50 mg/kg BW) intraperitoneally three times per week for 12 weeks. Body weight, food intake, visceral fat weight, uterine weight, serum lipid profile, glucose tolerance, insulin action on skeletal muscle glucose transport activity, and GLUT-4 protein expression were determined.

**Results:**

Prolonged ovariectomy resulted in dyslipidemia, impaired glucose tolerance and insulin-stimulated skeletal muscle glucose transport, as compared to SHAM. Treatment with *C. comosa *hexane extract and compound 049, three times per week for 12 weeks, markedly reduced serum total cholesterol and low-density lipoprotein levels, improved insulin sensitivity and partially restored uterine weights in ovariectomized rats. In addition, compound 049 or high doses of *C. comosa *hexane extract enhanced insulin-mediated glucose uptake in skeletal muscle and increased muscle GLUT-4 protein levels.

**Conclusions:**

Treatment with *C. comosa *and its diarylheptanoid derivative improved glucose and lipid metabolism in estrogen-deprived rats, supporting the traditional use of this natural phytoestrogen as a strategy for relieving insulin resistance and its related metabolic defects in postmenopausal women.

## Background

Insulin resistance syndrome is a complex metabolic abnormality of peripheral tissues in response to insulin. The main features of this syndrome include insulin resistance of skeletal muscle glucose metabolism, impaired glucose tolerance, compensatory hyperinsulinemia, essential hypertension, central obesity, and atherogenic dyslipidemia [1]. Postmenopausal women are at a greater risk for developing type 2 diabetes [2], a metabolic disorder characterized by glucose intolerance and insulin resistance. These metabolic conditions in postmenopausal women are usually accompanied by weight gain, obesity, and increased inflammation [3-5]. In addition, an increase in mortality associated with type 2 diabetes and cardiovascular disease has been directly attributed to insulin resistance and hyperinsulinemia [6]. Importantly, estrogen replacement can attenuate the increased risk for type 2 diabetes in postmenopausal women and improve whole body glucose metabolism [2,7], suggesting a critical role of estrogen in regulating glucose homeostasis. However, clinical studies on hormone replacement therapy in postmenopausal women have raised concerns about an increased risk of breast cancer and an unacceptable rate of unfavorable outcomes [8]. Therefore, the search for novel estrogen replacement strategies is an issue of increasing importance for postmenopausal women.

Phytoestrogens are naturally occurring non-steroidal plant-derived compounds with diverse structures which are found in many fruits, vegetables, and grains [9]. Studies on dietary phytoestrogens intake in postmenopausal women have indicated that high phytoestrogens intake is associated with a favorable metabolic cardiovascular risk profile, such as reduced plasma lipids and decreased aortic stiffness [10,11]. In addition, the use of phytoestrogens as an alternative intervention has been supported by evidences that incidence of menopausal symptoms and of breast and endometrial cancers are lower in Asian women who have a diet rich in soy products [12,13]. Plant-derived phytoestrogens are, therefore, considered to be an alternative remedy to prevent the development of metabolic defects in postmenopausal women.

*Curcuma comosa *Roxb. (Zingiberaceae) has been traditionally used in indigenous medicine in Thailand as a dietary supplement to relieve a variety of unpleasant peri- and postmenopausal symptoms. The hexane extract of *C. comosa *rhizome exhibited hypocholesterolemic [14,15] and anti-inflammatory activity [16,17]. Furthermore, treatment with *C. comosa *hexane extract improved the spatial memory of post-training [18] and effectively prevented bone loss in ovariectomized rodents [19]. The unique pharmacological actions of *C. comosa *underscore its therapeutic potential in relieving menopausal symptoms. Recently, the hexane extract of *C. comosa *and its isolated compound, a diarylheptanoid or (3*R*)-1,7-diphenyl-(4*E*,6*E*)-4,6-heptadien-3-ol (hereafter referred to as compound 049) have been demonstrated to have estrogenic activity [20,21]. Despite the long-term and widespread use of *C. comosa *extract, to date, no studies have been conducted to investigate its potential to reduce the risks of diabetes and cardiovascular disease in postmenopausal women. Therefore, the present study was undertaken to investigate the long-term (12 weeks) effects of *C. comosa *extract and compound 049 on metabolic alterations in rats with ovariectomy-induced insulin resistance. The results of this study provide mechanistic insight into the favorable effects of these compounds being used as an alternative strategy for relieving insulin resistance and its related metabolic defects in postmenopausal women.

## Materials and methods

### Chemicals

2-[1,2-^3 ^H] deoxyglucose (300 μCi/mmol), [U-^14 ^C]mannitol (0.8 μCi/mmol), and Ultima Gold™ scintillation cocktail were obtained from PerkinElmer Life Sciences (Boston, MA, USA), chemicals for the bicinchoninic acid (BCA) protein assay and bovine serum albumin were obtained from Sigma Chemical (St. Louis, MO, USA), Human R insulin (*Insulin, Human *Recombinant) from Eli Lilly (Indianapolis, IN, USA), ketamine from Gedeon Richter (Budapest, Hungary), xylazine from Farvet Laboratories (Bladel, The Netherlands), polyclonal anti-GLUT-4 antibody from Santa Cruz Biotechnology (Santa Cruz, CA, USA), and rabbit horseradish peroxidase-conjugated (IgG-HRP) secondary antibody and enhanced chemiluminescence (ECL) reagent from Cell Signaling Technology (Beverly, MA, USA). The glucose assay kit was obtained from Gesellschaft fur Biochemica und Diagnostica (Wiesbaden, Germany) and insulin radioimmunoassay kit from Linco Research (St. Charles, MO, USA). Pre-cast polyacrylamide gels were purchased from Pierce (Perbio Science, Cambrigde, England).

### C. comosa plant extract and compound 049 isolation

**Figure 1:**
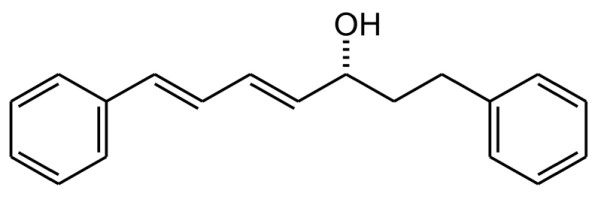
**Structure of (3*R*)-1,7-diphenyl-(4*E*,6*E*)-4,6-heptadien-3-ol: compound 049**.

*C. comosa *rhizomes were collected from the Kampaengsaen district, Nakorn Pathom province, Thailand. A voucher herbarium specimen has been deposited at the Department of Plant Science, Faculty of Science, Mahidol University, Bangkok (SCMU No. 300) [22]. Preparation of the *C. comosa *plant extracts were performed as previously reported [20]. Briefly, the rhizomes were sliced, dried, and ground to a powder. The powder was successively extracted with *n*-hexane and the solvent was removed *in vacuo*. For the hexane extract, a pale-brown viscous oil was obtained. This hexane extract was dissolved in corn oil for animal treatment. Characterization and standardization of the extract used in this study was conducted using reversed-phase HPLC (column: Prodigy ODS-3, 250 mm×4.6 mm, 5 μ, 100 Å; mobile phase: water-acetonitrile; detection at 260 nm), revealing a major diarylheptanoid (23.9%), (3*R*)-1,7-diphenyl-(4*E*,6*E*)-4,6-heptadien-3-ol (compound 049) (Figure 1). Compound 049 was isolated from the hexane extract as a major component by repeated silica gel column chromatography eluting with hexane/dichloromethane; each step utilized an increasing quantity of the more polar solvent.

### Animals and treatments

Animal procedures were approved by the Animal Care and Use Committee, Faculty of Science, Mahidol University, in accordance with the International Guiding Principles for Biomedical Research Involving Animals of Council for International Organizations of Medical Sciences (CIOMS). Adult female Sprague-Dawley rats (8-weeks-old), weighing between 180-200 g, supplied by the National Laboratory Animal Center, Thailand, were housed individually in 8 × 10 inch hygienic hanging metabolic cages at the Center of Animal Facilities, Faculty Science, Mahidol University. Animals were given standard rat chow and water *ad libitum*. The housing unit was maintained at 22-23°C with a 12/12 h light/dark cycle. Body weight was recorded every other day. The amount of food intake for each animal was measured over a 24 h period, and measurements were conducted at least 3 times per week. Rats were randomly assigned to either sham operation (SHAM, n = 8) or bilateral ovariectomy (OVX, n = 40). Animals were allowed to recuperate for 7 days after surgery. Ovariectomized animals were randomly divided into one of the following five groups: OVX-control (OVX), OVX receiving *C. comosa *hexane extract at a dose of 125 mg/kg body weight (BW) (OVX + C-125), OVX receiving 250 mg *C. comosa *extract/kg BW (OVX + C-250), OVX receiving 500 mg *C. comosa *extract/kg BW (OVX + C-500), and OVX receiving (3*R*)-1,7-diphenyl-(4*E*,6*E*)-4,6-heptadien-3-ol (compound 049) at dose of 50 mg/kg BW (OVX + 049). The *C. comosa*-treated animals were intraperitoneally administered 0.1 mL of the *C. comosa *hexane extract or compound 049 diluted in corn oil. SHAM and OVX-control rats received vehicle (0.1 mL corn oil). Treatment and vehicle were administered three times per week for 12 weeks.

### Determination of whole body insulin sensitivity

Oral glucose tolerance test (OGTT) was performed to assess insulin sensitivity at the whole-body level. Following a 12-week treatment period, an OGTT was performed on each animal. In the evening (1800 h) of the day before the test, rats were food-restricted to 4 g of chow. On the day of the test (0900 h), blood sample were collected from tail veins before and after glucose feeding (1 g/kg BW) at 15, 30, 60, and 90 min. Blood sample were mixed with EDTA and centrifuged at 13000 *g *at 4°C for 1 min. Plasma was kept at -80°C and used for glucose and insulin determination. The area under the curve for glucose (glucose AUC) or insulin (insulin AUC) was calculated. Subsequently, the glucose-insulin (G-I) index was calculated, as the product of the respective glucose and insulin AUCs, and is inversely related to whole-body insulin sensitivity [23].

### Assessment of muscle glucose transport activity

As skeletal muscle is the major tissue accounting for 70-85% of whole-body glucose disposal following a glucose challenge [24], insulin action on glucose transport activity was determined in skeletal muscle under basal and insulin-stimulated conditions using 2-deoxy-[^3 ^H]-glucose (2-DG) uptake. Five days after OGTT, animals were food-restricted as described above. At 0800 h, animals were weighed and anesthetized by intraperitoneal administration of a mixture of ketamine (50 mg/kg BW) and xylazine (10 mg/kg BW). Soleus muscle was isolated, divided into two portions of approximately 20-25 mg, and incubated for 60 min at 37°C in 3 mL of oxygenated Krebs-Henseleit Buffer (KHB) supplemented with 8 mM D-glucose, 32 mM D-mannitol and 0.1% radioimmunoassay-grade bovine serum albumin (BSA). One strip of soleus muscle was incubated in the absence of insulin, and the other strip in the presence of a maximally effective concentration of insulin (2 mU/mL). Flasks were continuously gassed with a mixture of 95% O_2 _and 5% CO_2 _throughout the incubation and transport study procedures. After the first incubation period, each muscle strip was rinsed for 10 min at 37°C in 3 mL of oxygenated KHB containing 40 mM D-mannitol, 0.1% BSA and insulin (for the muscle strip in the presence of insulin). Each muscle strip was then incubated for 20 min at 37°C in 2 mL of KHB containing 1 mM 2-[1,2-^3 ^H] deoxyglucose (2-DG), 39 mM [U-^14 ^C] mannitol, 0.1% BSA and insulin (for the muscle strip in the presence of insulin). At the end of the incubation period, muscle strips were removed and trimmed of excess fat and connective tissue, immediately frozen with liquid nitrogen and weighed. Frozen muscles were solubilized in 0.5 mL of 0.5 M NaOH, and 10 mL of scintillation cocktail was added. Specific intracellular accumulation of 2-DG was determined as previously described [25] using mannitol to correct for extracellular accumulation of 2-DG. Glucose transport activity was measured by determination of the intracellular accumulation of 2-DG (pmol/mg muscle wet weight/20 min). Contralateral soleus muscle was also removed, trimmed of fat and connective tissue, quickly frozen in liquid nitrogen, and used for GLUT-4 protein level determination.

### Serum lipid measurements

Following muscle dissection, blood was collected from the abdominal vein. Whole blood was allowed to clot and then centrifuged at 3000 *g *for 20 min to obtain serum. Serum levels of total cholesterol (TC), high-density lipoprotein cholesterol (HDL), and low-density lipoprotein cholesterol (LDL) were measured by enzymatic methods using an automated analyzer (Dimension RxL Max, DADE Behring, Marburg, Germany). Immediately after blood sample collection, visceral fat was collected from the superficial area covering the alimentary tract, and the uterus was removed and weighed.

### Muscle GLUT-4 protein content

Glucose uptake into skeletal muscle under insulin-stimulated condition is mediated by GLUT-4 protein [26]. To examine whether changes in insulin action on skeletal muscle glucose transport due to *C. comosa *treatment is associated with GLUT-4 protein expression, muscle GLUT-4 protein levels were evaluated. Portions of soleus muscle were homogenized as previously described [27]. These homogenates were used for total protein determination, solubilized, separated by electrophoresis using a 12% pre-cast polyacrylamide gel and transferred onto nitrocellulose paper. Protein blots were incubated with polyclonal anti-GLUT-4 antibody, GAPDH and subsequently with IgG-HRP secondary antibody. Protein bands were visualized by ECL on hyper film. Images were digitized and band intensities quantified using Image Master Totallab Software, version 3.0 (Amersham Pharmacia Biotech, Sweden).

### Statistical analysis

All values were expressed as the mean ± SE. Differences among groups were determined using a one-way analysis of variance (ANOVA) with Tukey's multiple comparison post-test using SPSS program version 16.0. A value of *P *< 0.05 was considered to be statistically significant.

## Results

### Body and tissue weight and food intake

**Figure 2:**
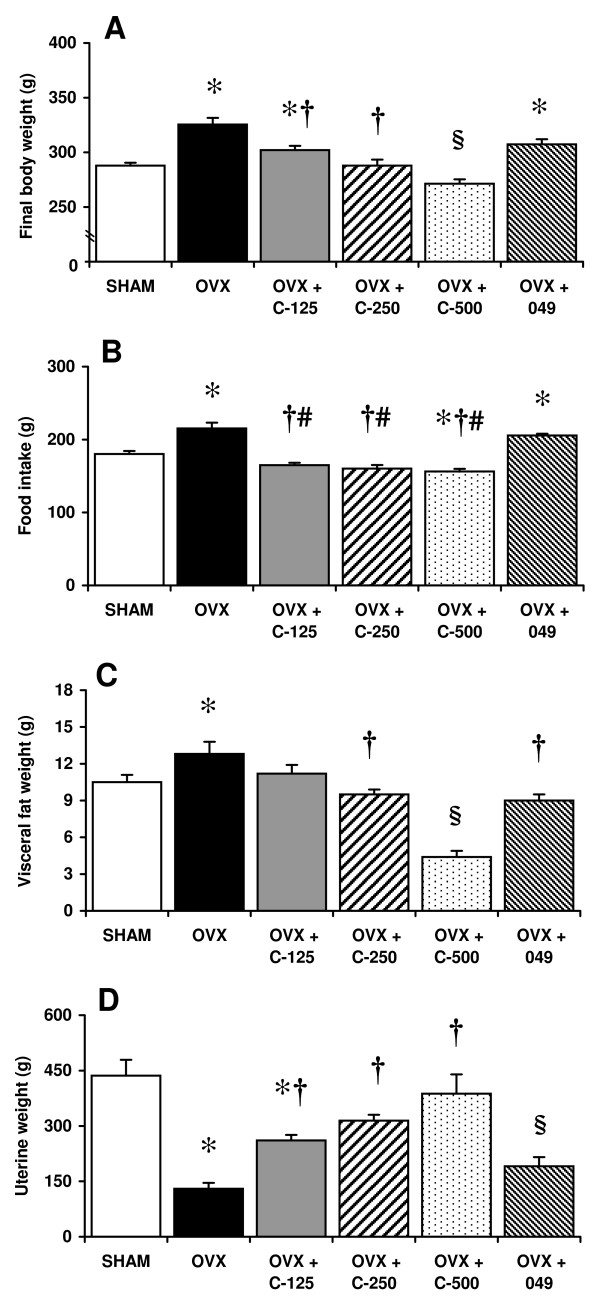
**Final body weight, food intake, visceral fat weight and uterine weight of sham operated control (SHAM) and ovariectomized (OVX) rats treated with or without *C. comosa *hexane extract at 125 mg (OVX + C-125), 250 mg (OVX + C-250), and 500 mg (OVX + C-500) per kg body weight or 50 mg of compound 049 per kg body weight (OVX + 049)**. Final body weight (A); accumulated amount of food intake (B); visceral fat weight (C); uterine weight (D). Values are reported as the mean ± SE for 8 animals/group. *P < 0.05 vs. SHAM group; **†***P *< 0.05 vs. OVX group; **#***P *< 0.05 vs. OVX + 049; §*P *< 0.05 vs. all other groups.

Twelve weeks following ovariectomy, OVX rats displayed significant weight gain compared to SHAM animals (Figure 2A). Body weight gain observed in OVX rats was significantly reversed by *C. comosa *extract treatment. Food intake, based on the accumulated average daily food intake throughout the 12-week experimental period, was significantly higher in OVX rats compared to either SHAM rats or OVX rats treated with *C. comosa *extract (Figure 2B). In contrast to the effects of *C. comosa *extract, compound 049 treatment did not affect body weight gain or food intake in OVX rats. Comparison of OVX rats with SHAM rats showed a 22% increase in visceral fat content, which was significantly reversed by treatment with either *C. comosa *extract or compound 049 (Figure 2C). The reduction in uterine weight in OVX rats was also partially recovered by treatment with either *C. comosa *extract or compound 049 (Figure 2D).

### Serum lipid profile

**Figure 3:**
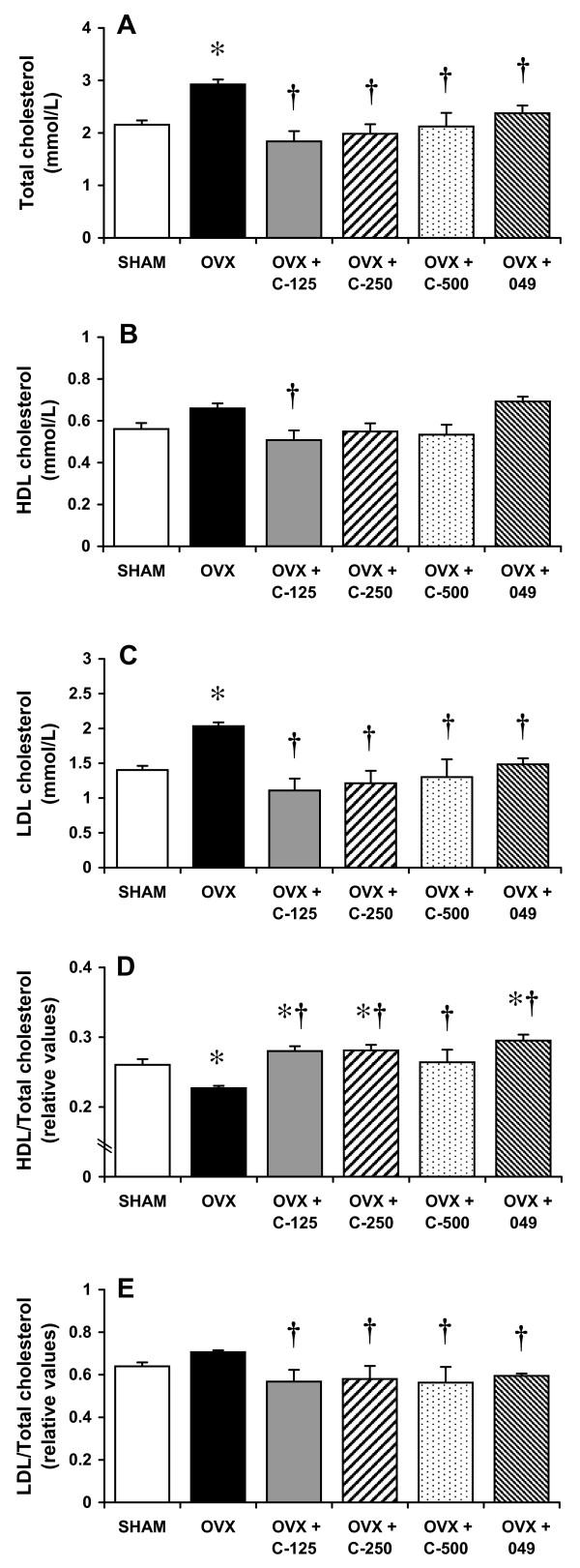
**Serum lipid levels of sham operated control (SHAM) and ovariectomized (OVX) rats treated with or without *C. comosa *hexane extract or compound 049**. Animals are as described in legend of Figure 2. (A) total; (B) high-density lipoprotein (HDL) cholesterol; (C) low-density lipoprotein (LDL) cholesterol; (D) ratio of HDL to total cholesterol (HDL/total); and (E) ratio of LDL to total cholesterol (LDL/total). Values are reported as the mean ± SE for 8 animals/group. *P < 0.05 vs. SHAM group; **†***P *< 0.05 vs. OVX group.

Compared to SHAM rats, OVX rats displayed a significant increase in TC (36%) and LDL (45%), but not HDL, whereas treatment with either *C. comosa *extract or compound 049 significantly reduced both TC levels (19-37%) and LDL levels (27-45%) (Figures 3A-C). *C. comosa *extract or compound 049 treatment resulted in a significant reduction in the LDL/TC ratio in OVX rats (Figure 3E). Interestingly, treatment with either *C. comosa *extract or compound 049 significantly improved the HDL/TC ratio when compared to the SHAM and OVX groups (Figure 3D).

### OGTT response

**Figure 4:**
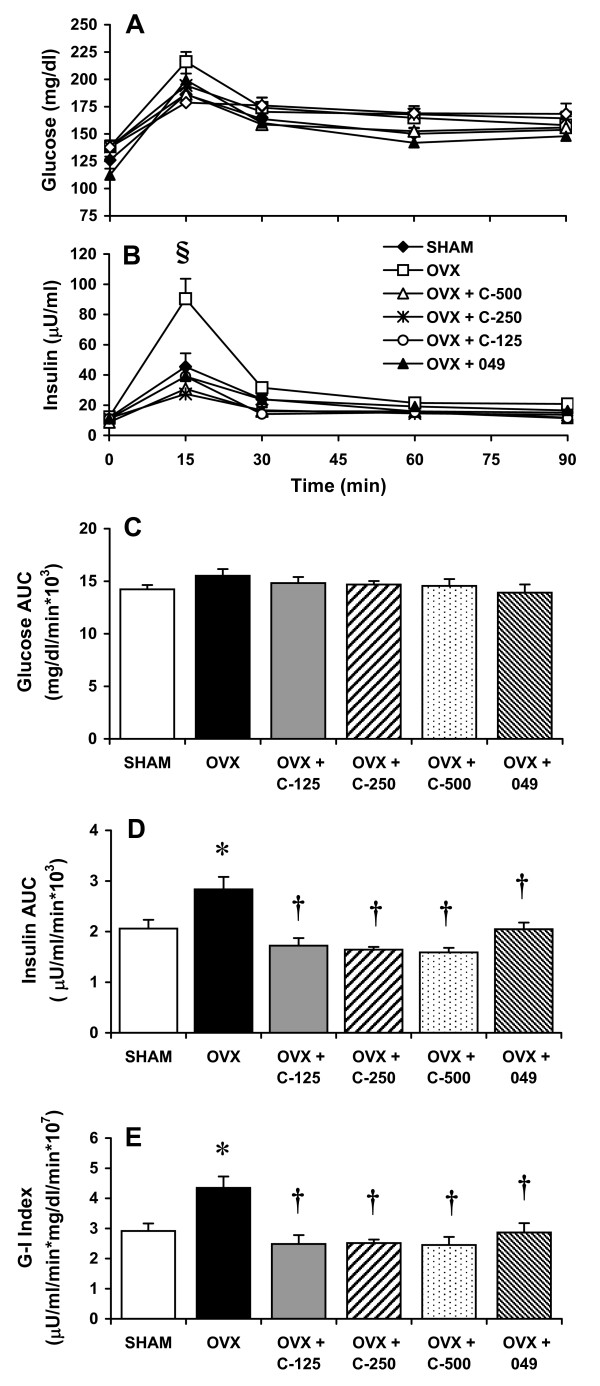
**Glucose tolerance tests of sham operated control (SHAM) and ovariectomized (OVX) rats treated with or without *C. comosa *hexane extract or compound 049**. Animals are as described in legend of Figure 2. Glucose (A) and insulin (B) responses; area under the curve (AUC) for glucose (C) and insulin (D); glucose-insulin (G-I) index (E). G-I index is the product of glucose AUC and insulin AUC for each individual animal. Values are reported as the mean ± SE for 7-8 animals/group. *P < 0.05 vs. SHAM group; **†***P *< 0.05 vs. OVX group; §*P *< 0.05 vs. all other groups.

Following glucose feeding (1 g/kg BW), there was no significant difference in plasma glucose levels among all groups throughout the experimental period (Figure 4A). However, 15-min after glucose challenge, plasma insulin levels in OVX rats were higher than those in other experimental groups (Figure 4B). Compared with the SHAM group, OVX rats displayed a significant rise in both insulin AUC (38%, Figure 4D) and G-I index (49%, Figure 4E). These elevated insulin AUC and G-I index values observed in the OVX group were reduced following treatment with either *C. comosa *extract (39-44%, Figure 4D) or compound 049 (28-34%, Figure 4E).

### Muscle glucose transport and GLUT-4 protein levels

**Figure 5:**
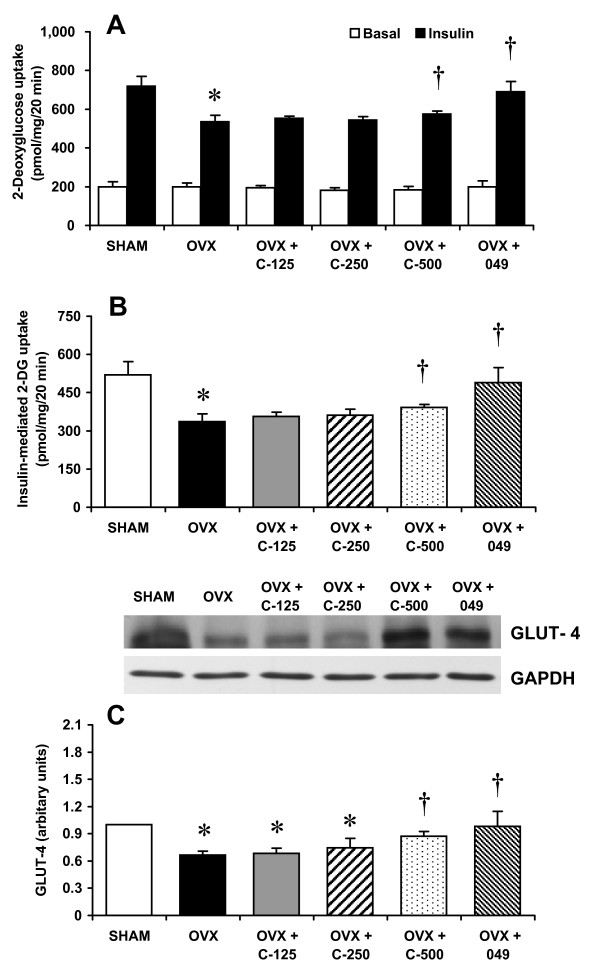
***In vitro *rate of 2-deoxyglucose uptake and GLUT-4 protein content in the soleus muscles of sham operated control (SHAM) and ovariectomized (OVX) rats treated with or without *C. comosa *hexane extract or compound 049**. Animals are as described in legend of Figure 2. (A) 2-deoxyglucose uptake in the absence (blank bar) and presence (filled bar) of insulin (2 mU/ml); (B) net increase above basal level for 2-deoxyglucose uptake due to insulin; (C) whole muscle GLUT-4 protein levels. GLUT-4 levels were normalized by GAPDH. Values are reported as the mean ± SE for 7-8 animals/group. *P < 0.05 vs. SHAM group; **†***P *< 0.05 vs. OVX group.

Skeletal muscle is the major tissue responsible for whole-body glucose disposal following a glucose challenge [24]. Thus, the effects of *C. comosa *and compound 049 treatment on insulin-stimulated glucose transport activity and on total GLUT-4 protein levels in skeletal muscle were studied. Basal 2-deoxy-[^3 ^H]-glucose (2-DG) uptake was comparable among the experimental groups (Figure 5A). Compared to the SHAM group, insulin-mediated 2-DG transport rates in soleus muscle isolated from OVX rats was reduced by 35% (Figure 5B). A significant improvement in insulin-mediated 2-DG uptake was observed in the OVX rats receiving either C-500 (16%) or compound 049 (45%) (Figure 5B). Twelve weeks following ovariectomy, total GLUT-4 protein levels in soleus muscle were reduced by 33% in OVX rats (Figure 5C). Treatment with either C-500 or compound 049 significantly restored GLUT-4 protein levels in soleus muscle by 17% and 47%, respectively (Figure 5C).

## Discussion

The present study demonstrated the protective effects of *C. comosa*, a novel natural phytoestrogen, against the risks of diabetes and cardiovascular disease in rats under estrogen-deprivation conditions. The evidence presented in this study corroborates the traditional use of *C. comosa *in relieving unpleasant symptoms in postmenopausal women including the metabolic defects. Moreover, this study indicates that these favorable effects observed following *C. comosa *extract treatment in the insulin-resistant condition can be attributed to multiple actions including its estrogenic and anti-inflammatory properties, and its diarylheptanoid derivative is at least accounted for the effects.

The role of estrogen in the regulation of glucose homeostasis has been shown previously. For example, postmenopausal women are at risk for increased incidence of obesity, type 2 diabetes, cardiovascular disease, and insulin resistance syndrome [5,28,29], whereas estrogen therapy reduces the incidence of insulin resistance and type 2 diabetes risks [30,31]. Insulin resistance develops when there is no estrogen in aromatase-knockout mice [32]. Hepatic insulin resistance and impaired skeletal muscle GLUT-4 expression are accounted for the impaired glucose tolerance and reduced insulin sensitivity in estrogen receptor alpha (ERα) knockout mice [33,34]. Due to ovarian hormone depletion, OVX rat becomes hyperphagic and gains weight with an increase in visceral fat accumulation [35,36]. In this study, adult female rats subjected to prolonged (12-week) ovariectomy were used to assess the metabolic impact of estrogen deprivation with *C. comosa *treatment in the postmenopausal state. We have previously reported that prolonged ovariectomy leads to the development of systemic metabolic conditions displaying key features of insulin resistance syndrome, such as increased visceral fat content, dyslipidemia, impaired glucose tolerance and decreased insulin-mediated glucose uptake in skeletal muscle [37]. These metabolic alterations in OVX rats were attenuated by estrogen replacement [37]. In this study, we observed that treatment of OVX rats with *C. comosa *extract, at all doses tested (125 to 500 mg/kg BW), results in significant improvements in whole body insulin sensitivity. Furthermore, a significant enhancement in insulin-stimulated skeletal muscle glucose transport and GLUT-4 expression was evident in the OVX group receiving the highest dose of *C. comosa *extract. Because the estrogenic activity of *C. comosa *hexane extract has been shown previously [20,21] and its estrogenic activity *in vivo *is confirmed here by our finding that the reduced uterine weight of OVX animals receiving *C. comosa *extract was reversed in a dose-related manner, it is possible that the beneficial effects of *C. comosa *on glucose metabolism in the estrogen-deprived state are attributed by the estrogenic activity of the extract.

Increasing evidence suggests that abdominal obesity and concomitant development of inflammation are major components of insulin resistance, and elevated levels of pro-inflammatory cytokines secreted from expanded adipose tissue can negatively modulate insulin signaling pathways [38]. Recently, it has been reported that treatment with *C. comosa *powder significantly reduced the expression levels of several pro-inflammatory cytokines in rabbits fed with a high-cholesterol diet [39]. Furthermore, *C. comosa *extract treatment reduces release of major pro-inflammatory cytokines in the microglia, peripheral mononuclear cells and a pro-monocytic cell line [16,17], highlighting the anti-inflammatory role of *C. comosa*. Thus the finding in this study, that treatment with *C. comosa *extract reduced the accumulated visceral fat content in OVX rats, suggests that *C. comosa *may improve insulin action by minimizing the source of pro-inflammatory cytokines. Our observation that estrogen-deprivation leads to atherogenic dyslipidemia is consistent with other earlier reports [40,41]. *C. comosa *treatment of OVX rats had favorable effects on plasma lipids, reducing the absolute levels of TC and LDL and the LDL/TC ratios while increasing HDL/TC ratios. The reduction in serum LDL cholesterol following treatment with *C. comosa *hexane extract here is consistent with our earlier studies, in which *C. comosa *extract decreased LDL and increased HDL cholesterol levels by increasing cholesterol excretion and elimination from the body via the feces [42]. Importantly, the decreased serum TC and LDL levels as a result of treatment with *C. comosa *powder in hypercholestorolemic animal were observed without causing liver toxicity [39]. Collectively, we provide evidence that treatment with *C. comosa *extract prevents the development of central adiposity, dyslipidemia, impaired glucose tolerance and skeletal muscle insulin resistance that occur as a result of estrogen deficiency. The mechanisms responsible for its favorable effects may be attributed by its estrogenic and anti-inflammatory properties.

In addition to demonstrating the beneficial effects of *C. comosa *extract treatment, we have further verified the identity of the active constituent of *C. comosa *extract and its metabolic effects. Compound 049, or (3*R*)-1,7-diphenyl-(4*E*,6*E*)-4,6-heptadien-3-ol, was found to be the major compound isolated from the *C. comosa *hexane extract [20]. Its estrogenic activity has been shown both in vitro [20] and in vivo [21], and its relatively weak estrogenic activity *in vivo *is confirmed here by our finding that the reduced uterine weight of OVX animals was only partially reversed by compound 049 treatment. In the present study, the comparable favorable outcomes were observed for serum lipid profiles, visceral fat accumulation, glucose tolerance and skeletal muscle glucose transport in estrogen-deprived rats treated with either compound 049 or *C. comosa *extract, whereas the body weight gain and energy intake in OVX rats were significantly reduced only in groups receiving *C. comosa *extract. This finding suggests that *C. comosa *extract and compound 049 modulated final body weights and the amount of food intake differently. Although it remains unknown how compound 049 acts to prevent the progression of insulin resistance in OVX rats, it possesses weak estrogenic activity which is consistent with the notion that the protective effect of compound 049 against the development of insulin resistance in the estrogen-deprivation condition may be related to other unidentified actions and independent to the estrogenic activity in the regulation of food intake and body weight. Based on the findings that compound 049 treatment leads to a significant improvement in Akt phosphorylation [43] and a reduction in expression of tumor necrosis factor-α [44] in aortic ring of OVX rats, we hypothesize that the mechanism underlying the enhanced whole-body insulin sensitivity and skeletal muscle insulin action in OVX rats by compound 049 treatment may be attributed to its action on tissues known to regulate glucose metabolism. While the opposing effects of the two subtypes of ER in the skeletal muscle have been reported, with ERα inducing and ERβ inhibiting GLUT-4 expression [34], it has been shown that compound 049 is an agonist for ER, and its biological action is ERα selective [45]. With our finding here that whole muscle GLUT-4 expression is increased following treatment with compound 049, it is likely that compound 049 selectively modulates estrogen receptor and improves insulin action in the skeletal muscle. The mechanisms by which compound 049 enhances whole-body insulin sensitivity and how it affects major metabolic tissues such as the liver and the pancreas are under investigation.

## Conclusions

This investigation indicates that improvements in glucose and lipid metabolism in estrogen-deprived rats manifest following 12-weeks of treatment with *C. comosa *extract, supporting the traditional use of this natural phytoestrogen as a strategy in relieving insulin resistance and its related metabolic defects in postmenopausal women. In particular, *C. comosa *extract effectively prevented the body weight gain, visceral fat accumulation, abnormal serum lipids, impaired glucose tolerance and skeletal muscle insulin resistance that occur as a result of estrogen deficiency. Importantly, these favorable effects of *C. comosa *extract on lipid profile, visceral fat content and insulin sensitivity are demonstrated to be due to its diarylheptanoid derivative.

## Abbreviations

C. comosa: *Curcuma comosa *Roxb; OVX: ovariectomy; Compound 049: (3*R*)-1,7-diphenyl-(4*E*,6*E*)-4,6-heptadien-3-ol; TC: total cholesterol; HDL: high-density lipoprotein cholesterol; LDL: low-density lipoprotein cholesterol; GLUT-4: glucose transporter 4.

## Competing interests

The authors declare that they have no competing interests.

## Authors' contributions

MP participated in the animal care and treatment, the glucose tolerance tests and in vitro glucose transport assay, and performed the statistical analysis. VS contributed to the conception and design of the experiments, the glucose tolerance tests and in vitro glucose transport assay, and drafted the manuscript. PP contributed to the conception and design of the experiments, and helped to draft the manuscript. AS contributed to the preparation and quantification of the *C. comosa *extract and compound 049. All authors read and approved the final manuscript.

## Pre-publication history

The pre-publication history for this paper can be accessed here:

http://www.biomedcentral.com/1472-6882/12/28/prepub
